# Mutation Screening of BRCA Genes in 10 Iranian Males with Breast Cancer

**Published:** 2016-05-09

**Authors:** Atieh Zorrieh Zahra, Sepideh Kadkhoda, Farkhondeh Behjati, Fatemeh Aghakhani Moghaddam, Azadeh Badiei, Fereidoon Sirati, Hossein Afshin Alavi, Morteza Atri, Ramesh Omranipour, Elahe Keyhani

**Affiliations:** 1 *Genetics Research Center-University of Social Welfare and Rehabilitation Sciences, Tehran, Iran.*; 2 *Cancer Institute- Department of surgery- Tehran University of Medical Sciences, Tehran, Iran.*; 3 *Pathology Ward-Day hospital,Tehran, Iran.*; # *These authors had equal contribution.*

**Keywords:** Male breast cancer, *BRCA* genes, human epidermal growth factor receptor, cytokeratin 5/6, estrogen receptor, progesterone receptor

## Abstract

Male breast cancer is a rare disease with an increasing trend. Due to limited information especially about the genetic basis of the disease in Iran and the lower age of its onset, the disease requires more attention. The aim of this study was to screen the male patients with breast cancer for BRCA mutations as well as tissue markers of estrogen receptor (ER), progesterone receptor (PR), human epidermal growth factor receptor (HER-2) and cytokeratin 5/6 (CK5/6). Ten Iranian males with breast cancer were selected regardless of their histologic subtypes, age and family history from patients referred to Mehrad, Day and Parsian hospitals in Tehran, Iran, during a two-year period. Paraffin blocks of the tumoral regions were tested for ER, PR, HER-2 and CK5/6 immunostaining. DNA extraction was carried out on the EDTA blood samples followed by Sanger sequencing. Immunohistochemistry results for ER, and PR were negative in 2 out of 10 patients, while the results of HER-2 and CK5/6 were negative in all the cases. A missense mutation in exon 18 of *BRCA1* and a nonsense mutation in exon 25 of in *BRCA2* were detected in one patient each. Both patients belonged to luminal A subtype. Despite the low number of patients in this study, it could be concluded that mutations in *BRCA1* and *BRCA2* occur in male breast cancer patients of luminal A subtype. The negative status of the tissue markers could not be used for the prediction of *BRCA* mutations.

Breast cancer is the most prevalent malignancy affecting women (1, 2). The disease is rare in males, accounting for 1% of all breast cancer cases (3-5) but recently a significant increasing rate from 0.86 to 1.08 cases per 100,000 populations has been reported (5-7). There are many studies reported from Iran on female breast cancer (FBC) but male breast cancer (MBC) still needs to be studied (8, 9). Unfortunately, in Iran few researches and reports about the prevalence of the disease are available (10). In Iran, MBC accounts for 0.65% of all cases of malignancy in men. There are 6674 incident cases of breast cancer diagnosed in Iran in 2007 of whom 3.26% were men (11). Due to its low incidence, there are few studies and limited information on this subject especially about the genetic etiology of the disease.

Furthermore, the average age of male breast cancer and female breast cancer in Iran is lower than developed countries (11), and men are usually diagnosed with breast cancer at more advanced stages often with lymph node metastasis (3, 12).

Several risk factors including advancing age, positive family history and mutations in susceptibility genes such as *BRCA1* and especially *BRCA2* are considered for the study of male breast cancer (10).

*BRCA1* and *BRCA2* are tumor suppressor genes, located on 17q21and 13q12 loci with 24 and 27 exons, respectively. These genes normally participate in the repair of damaged DNA by homologous recombination. Although *BRCA2* is the most clearly associated gene, *BRCA1* mutation is also considered in familial and even sporadic cases of male breast cancer (4, 5, 13). According to some studies, *BRCA2* mutations occur in 4-16% of male breast cancer patients. However, *BRCA1 *mutations are much less common and 0–4% of men with breast cancer carry these mutations (14). *BRCA1* mutations as a prognostic factor also have great importance, as carriers of these mutations have a poorer prognosis compared to other patients. Mutation in BRCA2 also has a special significance as the mutation carriers can be diagnosed with breast cancer at lower age and has a shorter survival (13). In comparison with *BRCA1* mutations, *BRCA2* mutations have major significance in MBC patients (4, 5, 13). Patients with mutation in *BRCA2* are predisposed to breast cancer and other malignancies such as pancreatic, prostate cancer and melanoma.

Molecular subtypes of MBC are similar to FBC (4, 11). According to several studies, MBC seems to be more hormone receptor positive (luminal A subtype) (3, 13, 14), than FBC. The main aim of this study was to screen the male patients with breast cancer for *BRCA1* and *BRCA2* mutations as well as the status of tissue tumor markers including ER, PR, HER-2 and basal marker (CK5/6). We also investigated the relationship between the *BRCA1* and *BRCA2* mutations and the tissue markers’ status.

## Materials and methods

**Patient selection and sampling**

In this cross- sectional study, 10 Iranian males with breast cancer were selected from Mehrad, Day and Parsian hospitals in Tehran, Iran, regardless of age, family history, histopathological subtypes and occupation. Consent forms were obtained according to the guidelines of the research ethics committee of the University of Social Welfare and Rehabilitation Sciences. 5-10 ml blood sample was collected into EDTA tube from each patient, and paraffin-embedded tissue block samples were prepared from the tumoral regions. Information about patients’ age, involved breast side, tumor size, histologic subtypes, status of lymph nodes’ involvement, surgical technique, tumor stage, tumor grade and family history of cancer was obtained from the patents’ medical files in the related hospitals. The median age for the breast cancer patients was 52.7 years (range from 38 to 72). The average tumor size was 2.03 cm (ranges 0.9 to 3.5). The histological types of all the tumors were invasive ductal carcinoma. Only one patient had documented family history of breast cancer with an affected mother. Six of the ten patients, showed the involvement of the left side, and in the other four cases the right side was involved.

**Histopathology**

Tissue sections were prepared, stained by hematoxylin and eosin (H&E) and then were studied by a pathologist to confirm the diagnosis and characterization of the tumor. Immunostainig was performed on 4 µm thickness sections. After incubation at 60°C and before proceeding with the staining protocol the slides were deparaffinized and rehydrated by xylene and ethanol (100%, 96% and 70%), respectively. Endogenous peroxidase activity was blocked with 5% hydrogen peroxide in methanol for 15 min. Heat- induced antigen retrieval with Tris-EDTA (pH=9) was done to break the methylene bridges and expose the antigenic sites in order to allow the antibodies to bind. Then slides were incubated with primary antibodies: 100 µl anti-human ER, PR, HER2, CK5/6 (Dako-Denmark). The secondary antibody or Envision was applied. The color was visualized by incubation with chromogen 3, 3’-Diaminobenzidine (DAB) for 5 min.. Then slides were placed in hematoxilin for 30 s and dehydrated in 70%, 96% and 100% (twice) ethanol and xylen (three times), one min. for each time, and finally were mounted. Positive and negative external controls were used to justify the test results. Interpretation of the results was performed using the classification method based on tissue tumor markers (Table 1).

**Table 1:** Classification of breast cancer based on tissue tumor markers.

**Subtypes**	**ER**	**PR**	**HER-2**
Luminal A	**+**	**+**	**_**
Luminal B	**+**	**+**	**+**
HER-2 Positive	**_**	**_**	**+**
Basal like	**_**	**_**	**_**
Normal breast like	**+**	**+**	**_**

For confirming HER-2 samples assessed by immunohistochemistry (IHC) as+2 score, FISH technique was performed (15). Routinely processed paraffin- embedded tissue sections (3 μm) were deparaffinized in fresh xylene and were rehydrated by ethanol (100%, 85% and 70%) and washed with distilled water. Slides were then pretreated with 0.2 M HCl for 20 min, and washed with distilled water. Slides were placed in a preheated 80°C pretreatment reagent (8% sodium isothiocyanate) for 30 min, rinsed in 2×SSC for 3 min. Protease digestion was accomplished by placing the slides in a pre-warmed (37 °C) protease solution (0.025% pepsin) for 30 min. Samples were then rinsed in distilled water for one minute, washed in 2×SSC for 5 min. and dehydrated in ethanol (70%, 85% and 100%). All slides were hybridized under identical conditions and with appropriate control tissue. 10 µl of probe was applied to the region of interest on the slide. A cover slip was placed and sealed with rubber cement. The slides were denatured on a hot plate at 80 °C for 5 min. and incubated overnight at 37 ºC in a humidified chamber. After hybridization, the rubber cement was removed. When cover slips did not come off easily, slides were washed in 2×SSC / 0.1% igepal for 2 min, then washed in 0.4×SSC/ 0.3% igepal at 72 °C for 2 min and 2×SSC/ 0.1% igepal for 1 min. Slides were dehydrated in ethanol (70%, 85% and 100%) and counterstained by applying 10 µl of 4,6-diamidino-2- phenylindole dihydrochloride (DAPI) and a glass cover slip was placed on the slide. At least 20 cells were scored in each preparation, and the total number of *HER2* and *CEN17* signals in each cell was scored using a fluorescence microscope.

The basis of IHC scoring according to American society of clinical oncology/college of American pathologists (ASCO/CAP) guideline is as follows: As HER-2 is localized in membrane of tumor cells, IHC scoring of 3+, 2+, 1+ and 0 are defined as a strong complete, weak or moderate, faint or incomplete membrane, and no stain or membrane stain in less than %10 of tumor cells. The localization of ER or PR antigens is nuclear, in immunostaining. So staining in more than 1% of the tumor cell nuclei means positive expression and less than 1% staining reveals negative result. Concerning HER-2 staining, *HER-2*/*CEN17* ratio< 1.8= negative, ratio= 1.8-2.2 means equivocal and ratio> 2.2 means positive.

**Gene sequencing**

Genomic DNA was extracted from the blood samples using salting out method. The quality and quantity of DNA was determined by reading the optical density of the sample at 260 nm and 280 nm. Primers for *BRCA1* and *BRCA2* genes were designed by primer 3 and UCSC genome browser. Self-dimers, heterodimers and the melting tempera-ture of the primers were also checked by oligo analyzer site (https:// www. idtdna.com/ calc/ analyzer).

In *BRCA1*, exon 10 and 24 were separated into 6 and 3 fragments, respectively. In *BRCA2*, exons11, 10, and 27 were separated into 9, 3, and 2 fragments respectively. DNA was amplified by polymerase chain reaction (PCR) technique.

Mutation analysis was performed by direct DNA sequencing of all the exons of *BRCA1* and *BRCA2*. The partial flanking intronic sequences were obtained using codon code aligner and Gene runner softwares

## Results

**Immunohistochemical analyzes**

In this study, we screened 10 Iranian men with breast cancer in order to find any mutation in *BRCA* genes. On the other hand, tissue tumor markers (ER, PR, HER-2 and CK5/6) status, in related tissues of the patients was assessed. Analysis of the stained sections confirmed the presence of the tumoral tissue.

**Fig 1:**
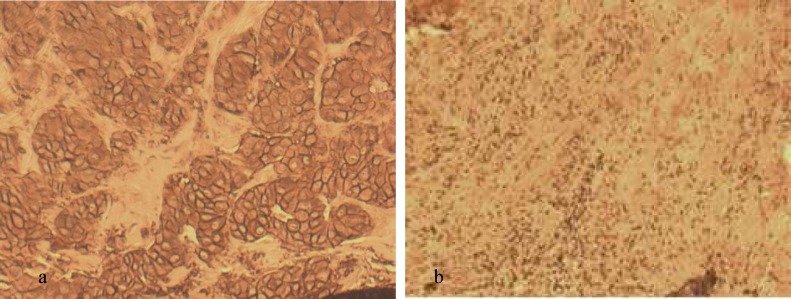
Immunohistochemical staining for HER2. Different staining patterns are seen in two patients. a: positive control with strong continuo-us membrane staining which is a good example of a 3+ positive tumor (3+); b: negative HER2 with no staining in membrane (0). Original magnification × 400; scale bar: 100 μm.

**Fig 2:**
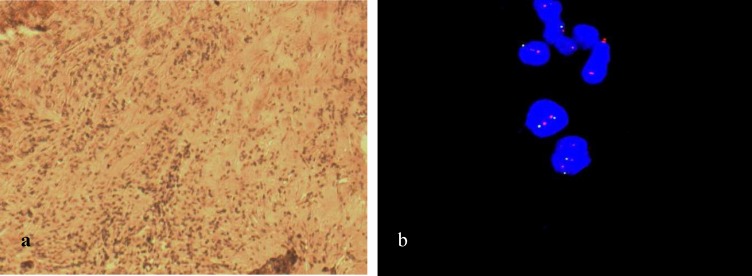
Immunohistochemical staining and FISH for HER2. a: HER2 with score 2 by IHC (borderline).There is a moderate membrane staining pattern. In this case, tumoral tissue was tested by FISH to confirm the diagnosis. Original magnification×400; scale bar: 100 μm. b: FISH staining for evaluating IHC for HER2 score 2 patient revealed an absence of HER2 amplification. Red signal (Text-Red): HER-2, Green signal (FITC): chromosome17 centromere. For evaluating FISH results, 20 cells from 2 tumor regions were counted; scale bar: 10 μm.

**Fig 3:**
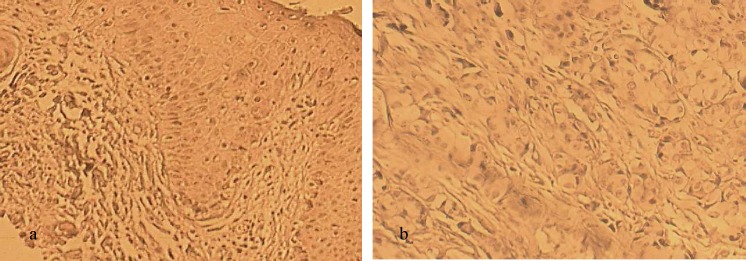
Immunohistochemical staining for CK5/6. a: positive control with cytokeratin 5/6 expression; b: negative CK5/6 with no expression of CK5/6. Original magnification × 400, scale bar: 100 μm.

Results of IHC technique for CK5/6 were negative for all patients. For ER and PR markers 8 out of 10 cases were negative and 2 out of 10 had positive markers. The score of IHC for HER-2 marker was +2 for 3 patients, and FISH technique was carried out to confirm the results. Average ratio of *HER-2*/*CEN-17* for these patients was 1.1. Therefore, the amplification of *HER-2* in these samples was negative. The 7 other patients had a score lower than +2 in IHC evaluation and were considered also as negative for Her 2. The results for IHC as well as FISH stainings for *HER2*, followed by IHC for CK5/6, are shown in Figures 1- 3.

**Mutation analysis**

Following PCR amplification and sequencing of *BRCA1* and *BRCA2*, in 10 males with breast cancer, their variants and polymorphisms were detected. Further information, including the exon number, base changes and frequency of these variants in 10 patients, are shown in Tables 2 and 3 for the *BRCA1* and *BRCA2*, respectively.

**Fig 4:**
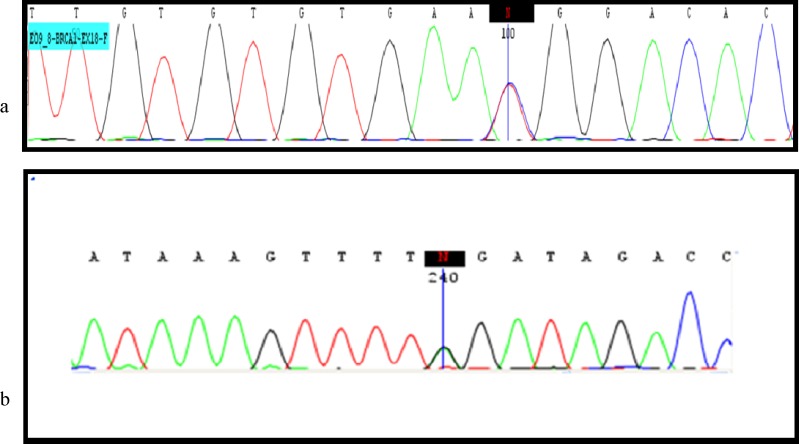
*BRCA *sequencing results. a: sequence graph of the region surrounding the exon 18 of *BRCA1 *c.5158C>T mutation in the patient (5158C/T heterozygous); b: DNA sequencing of *BRCA2 *exon 25 identified a heterozygous *BRCA2 *mutation (c.9317G>A) in the patient.

**Table 2:** Polymorphisms and variants in *BRCA1* gene.

**Variants**	**Exon**	**Base change**	**Frequency n= 10 (100%)**
rs1799949	10	C>T	0.7
rs16940	10	T>C	0.6
rs1799950	10	A>G	0.1
rs4986850	10	G>A	0.2
rs799917	10	C>T	0.5
rs16941	10	A>G	0.4
rs16942	10	A>G	0.5
rs80357396	10	A>T	0.1
rs8176318	24	G>T	0.5
rs12516	24	C>T	0.6
rs799923	7	G>A	0.2
rs1060915	12	T>C	0.7
rs1799966	16	A>G	0.6

**Table 3:** Polymorphisms and variants in *BRCA2* gene**.**

**Variants**	**Exon**	**Base change**	**Frequency n=10 (100%)**
rs17999432	10	G>A	0.2
rs766173	10	A>C	0.4
rs1801439	10	A>G	0.4
rs144848	10	A>C	0.3
rs1801499	11	T>C	0.4
rs1801406	11	A>G	0.2
rs1799944	11	A>G	0.4
rs543304	11	T>C	0.5
rs206075	11	A>G	1
rs80358755	11	A>G	1
rs206076	11	A>G	1
rs169547	14	T>C	1
rs1799955	14	A>G	0.2
r rs80359157	23	C>T	0.1

A missense mutation was detected in exon 18 of *BRCA1* at position c.5158C>T (Figure 4a, Table 4). This mutation was evaluated by Mutation Taster, Mutation Assessor, SIFT, PROVEAN, POLY PHEN and ENSMBLE softwares and was confirmed as a cause of disease or pathogenic variant by BIC and HGMD databases. In another patient a nonsense mutation was found in exon 25 of *BRCA2* at position c.9317G>A (Figure 4b, Table 4).

This mutation was evaluated by BIC database, mutation taster site and HGMD database as a pathologic mutation Interestingly, in both patients with *BRCA* mutation, IHC results and tumor markers status (ER, HER-2 and CK5/6) were identical to other patients, e.g. positive for ER and negative for the two other markers. Furthermore, none of the patients with *BRCA* mutation had a family history of the disease. The informations about patients including age, stage, lymph node status, degree and tumor markers’ status assessment along with the type of related mutation in *BRCA* genes are presented in Table 5.

**Table 4:** Results of the evaluation of *BRCA* sequencing

Gene	Exon	Mutation	AA change	Mutation type
*BRCA1*	18	c.5158C>T	Arg1720Trp	missense
*BRCA2*	25	c.9317G>A	Trp3106Ter	stop codon

**Table 5:** Pathologic features, tumor markers assessment and mutation screening of male breast cancer patients

**Patient**	**Age**	**Side**	**Stage**	**Grade**	**Axillary Lymph Node Involvement**	**ER**	**PR**	**HER-2**	**CK5/6**	***BRCA1***	***BRCA2***
01	44	Left	І	Ш	+	+	+	_	_	_	_
02	72	Left	Шb	Ц	_	_	_	_	_	_	+
03	48	Left	Цa	Ц	_	+	+	_	_	_	_
04	57	Right	І	Ш	+	+	+	_	_	_	_
05	49	Left	Цa	Ц	+	+	+	_	_	+	_
06	44	Left	І	Ц	Not available	+	+	_	_	_	_
07	52	Right	Ц	Ц	Not available	+	+	_	_	_	_
*08	38	Right	Ц	Ц	_	+	+	_	_	_	_
09	65	Right	І	Ц	_	+	+	_	_	_	_
10	57	Right	І	Ш	_	_	_	_	_	_	_

* The patient has a family history of breast cancer.

## Discussion

In this study, 10 males with breast cancer were screened for mutations in *BRCA1* and *BRCA2*. Besides, the patients were characterized for immunohistochemical features such as ER, PR, Her-2, and CK5/6 tissue tumor markers. In one patient, mutation in exon 18 of *BRCA1* was detected. Furthermore, another patient has a nonsense mutation in exon 25 of *BRCA2*. Moreover, several variants and polymorphisms associated with *BRCA1* and *BRCA2* were found, also some features correlated to those, such as the exon number, base changes and frequency of these variants, were summarized in Tables 3 and 4. As the purpose of the current study was mutation screening in *BRCA1* and *BRCA2*, associated variants were identified and assessed in order to note whether they were disease causing. Then mutation screening was followed until a mutation was found and confirmed by some known softwares and databases. Results of IHC technique for HER-2 and CK5/6 markers of all patients and ER and PR markers for two patients were negative.

Previous studies have indicated that mutation in *BRCA2* in MBC is more common than *BRCA1*, but our findings could not conclude such results due to the small sample size. According to a similar study by Deb and his colleagues in Australia in 2012, most of MBC patients (89.7%) were classified in luminal subtypes and a minority were in HER-2 and basal categories. The rate of mutation in *BRCA2* was higher and in *BRCA1* was lower compared to FBC (6).

Based on other studies, positive status of ER and negative status of CK5/6 tissue markers followed by negative status of HER-2, concluded luminal A molecular subtype of MBC. The status of the studied tissue tumor markers could not predict the existence of mutation in *BRCA* genes.

Some studies have only focused on *BRCA1* or

*BRCA2* or IHC features (16, 17), and therefore concurrent review of *BRCA1/2* mutational screening together with tumor markers have rarely been reported (17). Several studies based on concurrent investigations of *BRCA* genes and tumor markers performed only in FBC patients and outside Iran, showed results similar to the present study (18). While this project is a pioneer study based on genetic and histopathologic aspects of MBC in Iran, there is still a great need to study the genetic basis of MBC.

It should be mentioned that the main problems of this project were: “case finding” and “tiny tissue samples”. Considering the low prevalence of the disease around the world, especially in Iran, despite searching four hospitals to find males with breast cancer, and due to the problems such as lack of cooperation of the patients and their families, finally a small population of only 10 qualified cases were allocated for the study. According to the rarity of MBC and its increasing rate in the world, it seems to be necessary to perform studies with larger sample size. We believe that general education for the early diagnosis of breast cancer in males in order to increase their awareness of this disease, and also promotion of the quality of diagnostic methods in Iran are necessary. 
